# Prevalence and factors associated with intestinal parasitic infection among under-five children in and around Haro Dumal Town, Bale Zone, Ethiopia

**DOI:** 10.1186/s12887-019-1731-0

**Published:** 2019-10-27

**Authors:** Eshetu Gadisa, Kefiyalew Jote

**Affiliations:** Menelik Medical and Health Science College, Kotebe Metropolitan University, P.O.Box:3268, Addis Ababa, Ethiopia

**Keywords:** Selective deworming, Parasite, Geo-helminthes

## Abstract

**Background:**

Intestinal parasitic infection is diversified illness and diseases caused millions morbidity among under-five children lives in developing countries particularly vulnerable rural communities. Deworming coverage in such community is low. The aim of this study was to determine the prevalence and associated risk factors of intestinal parasitic infections (IPIs) among under-five children live in and around Haro Dumal Town.

**Methods:**

Community-based cross-sectional study was conducted in 561 randomly selected under-five children from June to August, 2018. The stool samples were collected and examined by basic parasitological techniques. Data related to socio-demographic and risk factors were collected using a self administered questionnaire. Statistical data analysis was done using SPSS version 21 and the bivariate and multivariate logistic regression used to compute the association between variables. *P*-value of < 0.05 was statistical significance.

**The results:**

Of the 561 total under-five children, 216 (38.5%) were found to be infected with intestinal parasites. *E.histolytica/dispar* (15.3%) was the most prevalent parasite, followed by hook worm (14.4%) and *T.trichuria (*13.9%). Regarding risk factors, geo-phage [(AOR = 4.7; 95%CI: 2.0–10.4), *P* < 0.001], tungiasis [(AOR = 3.1; 95%CI: 1.1–6.6), P < 0.001], eating raw vegetable [(AOR = 1.3; 95%CI: 1.4–3.3), P < 0.001] were significantly associated with intestinal parasitic infections.

**Conclusion:**

Intestinal parasitic infections **(**IPIs) were found to be highly prevalent in the study area. Hence, improving sanitation, controlling ecto-parasite such as tungiasis, provision of safe water and successful mass-deworming are important.

## Background

Intestinal parasitic infections are illness and diseases caused by helminths and protozoan [1]. These infections have been occurring predominantly in developing countries and remain a major public health problem with vast socioeconomic devastation among vulnerable rural communities [2–4]. According to the WHO report in 2014, more than 3.5 billion people were infected with intestinal helminths mainly by *Taenia saginata, S.stercoralis, H.nana*, *A. lumbricoides, T.trichiura* and hookworms [5]. From helminthic infections that are grouped under geo-helminthic; *A. lumbricoides, T.trichuria* and hook worm are widely distributed in sub-Saharan Africa, the Americas, China and East Asia [6, 7]. Many studies showed that more than two billion people were infected by geo-heminthic in worldwide. Two third (2/3) of African countries had high risk areas with prevalence of more than 50% [8]. On the other hand, neglected intestinal protozoans like *E. histolytica/dispar*, *G. lamblia* and other coccidian are triggering millions morbidity and mortality among children, pregnant women and immune-compromised people [9, 10].

People are infected through ingestion of infective stages (eggs, cyst,) or skin penetration by larvae stage of the parasites with contaminated soil, water and under cooked meat and/or vegetables [11–13]. The occurrence of parasitic infections varies with the level of sanitation, water, environment, climates, host and parasitic factors [14]. Generally, they are more predominant illnesses or diseases in the tropics and sub-tropics than temperate climate [15]. Overall, the intestinal parasites could be causes of most illnesses that range from simple and asymptomatic (fever, abdominal pain, weight loss, diarrhea, anemia, malnutrition, mal-absorption, peri-anal irritation and cough) to complex and life threatening problems such as hepatomegaly, obstruction, appendicitis and pancreatic obstruction, seizures and hydrocephalus [16, 17]. The infections may lead to impaired growth, stunting, physical weakness and low educational performance of infected children. It is also imposes large health and socioeconomic burden on societies [17].

The cost-effective anti-helminthic drugs fail to achieve the desired results due to poor sanitations, inaccessibility of safe water, inadequate waste drainage system and favorable climate for transmission of the intestinal parasitic infection in Africa [17]. As a result, pregnant women and children are suffering from parasitic infections give rise to economic crunches, poverty and malnutrition. This has a negative impact on attempts to reduce the maternal and infant mortality rates in the developing countries in general and Sub-Sahara Africa in particular [18]. In line with this, many countries, including Ethiopia have been launching selected deworming programs to control intestinal geo-helminthic infections among preschool-age children to reduce their morbidity and mortality [7, 18].

Despite the presence of selective deworming drugs for under-five children and promotion of health education through health extension workers, intestinal parasitic infections are the leading cause of morbidity and mortality. This is impeding the efforts to achieve sustainable development goals [19]. Poor sanitation, untreated water, inadequate drainage system and poor healthcare system accelerated socioeconomic devastation and hinder healthcare service provision. Effective poverty reduction programmers and promotion of deworming could reduce intestinal parasite carriage [17–19]. However, there has been need thoroughly evaluate the effectiveness and efficiency of the treatment, prevention and control methods, and risk factors and the way of mitigating of intestinal parasitic infections vulnerable rural communities. Still, there is low selective deworming coverage among under-five children for prevention of geo-helminthes in the most developing countries [20]. Therefore, determine the prevalence and associated risk factors with intestinal parasitic infection are urgently needed for vulnerable communities live in rural areas.

## Methods

### Study area

This study was conducted in and around Haro Dumal Town, Berbere Woreda, Bale Zone. Haro Dumalis located 290 km away from Robe (Zonal Town) in the Southeastern direction. The town lies at an elevation of 1800 m above sea level and it is located at coordinates Latitude; 6° 39′ 59.99“ N and Longitude; 40° 09’ 60.00” E. The town lies at the foot mountain Gara Jarti (West), Tullu Rifensa (South) and Gara-Bale. The hills serve as part of the watershed for the Dumal River and Badadicho River. The town is characterized by a plane landscape with pockets of hills and slope landscape. The well, river, pipe water, stream and rain water are the major source of water used for drinking and other purpose. Some of the communities have pit latrine that closed to water sources while others defecate in the nearby rivers. As a result, the flood during rainy season increases sanitation problem.

### Study design and period

A community-based cross-sectional study was conducted to determine prevalence and associated risk factors of intestinal parasitic infections among under-five children living in and around Haro Dumal town, Bale Zone from June to August, 2018.

### Source of population and study population

The sample was allotted identified proportionally from all kebeles using systematic random sampling at an interval of 15 households live in and round the town. Structured questionnaire was adapted by reviewing different literatures [1, 3, 12]. The questionnaire covers socio-demographic characteristics, knowledge about the parasite route of transmission, prevention and control. The data were collected by face to face self administered questionnaire from each family. We selected only one child from each household where there are two or more eligible family members. The questionnaire was translated to the local language (Afan Oromo) and retranslated back to English to check its consistency by language expertise. Pretest was done on 5% of the sample before the actual study. On top of this, the investigators checked for the collected data and samples on daily bases.

### Sampling size and sampling procedure

The sample size was determined by using single population proportion formula using an assumption of 95% confidence interval, 0.05 margin of error and considering 10% non-response rate. Thus, the final sample size was 561.

### Sample collection for laboratory

Sample collection, process and transportation technique was adapted from clinical and laboratory standard institute guidelines used for surveillance of parasitic infection. The stool sample was examined for the presence of different stages of parasites (adult, trophozoite, larvae, cysts and ova) using direct wet mount and modified formal-ether sedimentation techniques [14].

### Data management and analysis

Data were entered in to Epi data version 3.1 and was exported to SPSS version 21 for analysis. Descriptive analysis was applied to determine the proportion of intestinal parasites. Bivariate and multivariate analysis was employed to identify independent predictors of parasites.

### Ethical clearance

Ethical issue was approved by Kotebe Metropolitan Univeristy, Menelk Medical and Health Science College, Depetment Medical Laboratory Science and the ethical review committee. Support letter was obtained from the college to health centers. Informed written consent was obtained from their mother’s after clarifying the aim of the study. The respondents were informed about the right to respond fully or partially to the questionnaire. All data given by the respondents were kept confidential and used for research purposes only and confidentiality was maintained by omitting the name of the respondents. Other concerns related to sample collection, laboratory processing and discarding leftover specimens were as recommended for infection prevention and control strategies standard.

## Results

### Socio-demographic characteristics

A total of 561 randomly selected under-five children living in and around Haro Dumal Town were included in the study with an average age of 3.29 years. Fifty six percent of them were female. 16% of mothers were educated above the primary and 21.4% of them didn’t know the way of transmission of intestinal parasites. Regarding mother’s occupation, unemployed accounts 405(72.2%) followed merchant 151(26.9%). The mean ± standard deviation monthly family income (USD) was 43.2 ± 11.5 with a median of 31.3 (Table 1).

**Table 1:** Socio-demographic characteristics and type of infection of under-five children live in and around Haro Dumal Town, Bale Zone, Ethiopia, 2018

Characteristics	Frequency No (%)	No positive IPI (%)	*P*-value
Age			
6 months—1 year	82 (14.6)	17 (7.9)	0.03
1–2 years	97 (17.9)	29 (13.4)	
2–3 years	113 (20.1)	36 (16.7)	
3-4 years	117 (20.9)	53 (24.5)	
4–5 years	152 (27.1)	81 (37.5)	
Gender			
Male	242 (43.2)	107 (49.4)	0.305
Female	319 (56.8)	109 (50.6)	
Mother educational status			
No formal education	171 (30.5)	93 (43.0)	0.08
Elementary school	301 (53.7)	57 (26.4)	
High school	81 (14.4)	49 (22.7)	
Certificate and above	8 (1.4)	17 (7.9)	
Mother occupation			
Housewife/ unemployed	405 (72.2)	131 (60.6)	0.86
Employee	5 (0.9)	3 (1.4)	
Merchant	151 (26.9)	82 (38.0)	
How a child gets infected with intestinal parasites?			
Contaminated food	161 (28.7)	–	0.29
Drinking dirty water	179 (31.9)	–	
Drinking raw milk	101 (18.0)	–	
Evil eye	79 (14.1)	–	
I do not know	41 (7.3)	–	
Does tungiasis contribute to IPI?			
Yes	489 (87.1)	93 (43.1)	<0.01
No	56 (10.0)	5 (2.3)	
I do not know	16 (2.9)		
Monthly family income (USD)			
<18	250 (44.6)	111 (51.4)	0.91
19–54	299 (53.3)	103 (47.7)	
>55	12 (2.1)	2 (0.9)	

*P*-value of X^2^ test of the trends, *IPI* Intestinal parasitic infection

### Prevalence of intestinal parasites

This study revealed that seven species of intestinal helminthes and two species of protozoan were identified in the stool samples. Overall the prevalence of intestinal parasites was 38.5%. The hook worm was the predominant intestinal helmintic parasite with prevalence of 14.4%. *T. trichiura* and *A. lumbricoides* were frequently detected intestinal nematodes with a prevalence of 13.9 and 13.4% respectively. *H.nana*, and *T. saginata* were the detected cestodes with a prevalence rate of 25(11.6%) and 13(6.0%) respectively. Moreover, the prevalence of two most common intestinal pathogenic protozoan *E. histolytic* and *G. lamblia* infections were 33(15.3%) and 22(10.2%) (Table 2). About 76.9% of under-five children were infected with mono-parasites. Of which, 46.8% were intestinal nematodes followed by protozoa (16.7%). On the other hand, 23.1% of the parasitic infections were found to be poly-parasitism (Table 3).

**Table 2:** Bivariate analysis of factors associated with IPI among under-five children live in and around Haro Dumal Town, Bale Zone, Ethiopia, 2018

Risk factors	Total (%)	Positive IPI	Crude OR 95%CI	*P*-value
Eat undercooked/ raw vegetable				
Yes	269 (48.0)	125 (46.5)	1.6 [0.9–3.7]	0.001
No	292 (52.0)	91 (31.2)	1.0	
History of excessive crying				
Yes	317 (56.5)	170 (78.7)	2.4 [1.0–4.4]	0.01
No	244 (43.5)	46 (21.3)	1.0	
Finger nail trimming				
Yes	349 (62.2)	141 (65.3)	1.9 [1.1–2.6]	0.01
No	212 (37.8)	75 (34.7)	1.0	
Geo-phage				
Yes	356 (63.5)	115 (53.2)	5.2 [2.0–11.3]	0.01
No	205 (36.5)	101 (46.8)	1.0	
Latrine care				
Mother	117 (20.9)	58 (26.9)	0.9 [0.5–6.6]	0.61
Self	441 (78.6)	157 (72.7)	4.7 [1.6–12.5]	0.001
Others	3 (0.5)	1 (0.4)	1.0	–
Protective shoe wear habit				
Yes	124 (22.1)	84 (38.9)	1.0	–
No	437 (77.9)	132 (61.1)	2.6 [2.2–7.3]	0.01
Child infected Tungiasis/month				
1 time	82 (16.8)	3 (3.2)	–	–
2 times	271 (55.4)	43 (46.2)	1.9 [0.2–3.5]	0.01
≥3 times	136 (28.8)	47 (50.6)	5.6 [0.3–7.63]	0.001
Type of intestinal parasite				
Protozoa	*E.histolytica*	33 (15.3)	–	55/216 (25.5%)
	*G.lambia*	22 (10.2)		
Nematodes	*Hook worm*	31 (14.4)	–	123/216 (56.9%)
	*T.tricuria*	30 (13.9)		
	*A.lumbricoide*	29 (13.4)		
	*Evermicularis*	19 (8.8)		
	*S.stercolaris*	14 (6.5)		
Cestodes	*H.nana*	25 (11.6)	–	38/216 (17.6%)
	*T.saginata*	13 (6.0)		

*IPI* intestinal parasitic infection, *OR* Odd Ratio, *CI* Confidence Interval, *P*-value of X^2^ test of the trends

**Table 3:** Proportion of cases with mono-parasitism and poly-parasitism of IPI among under-five children live in and around Haro Dumal Town, 2018

Type of infection	Classification of parasites	No cases	Percent (%)
Mono-parasitism	Protozoan	36.0	16.7
	Nematodes	101.0	46.8
	Cestodes	29.0	13.4
Poly-parasitism	Protozoan and nematodes	20.0	9.3
	Nematodes and cestodes	15.0	6.9
	Protozoan and cestodes	5.0	2.3
	Protozoa, nematodes and cestodes	10.0	4.6
Total		216	100.0

*IPI* intestinal parasitic infection

### Factors associated with intestinal parasites

As data obtained from their mothers, the risk factors such as eating raw/undercooked/ vegetable [(OR = 1.6; 95% CI:0.9–3.7),*P* = 0.001],habit of finger nail trimming regularly[(OR = 1.9; 95% CI: 1.1–2.6,*P* = 0.01)], geo-phage[(OR = 5.2;95% CI:2.0–11.3),P = 0.01], latrine care by themselves [(OR = 4.7; 95% CI:1.6–12.5), P = 0.001], no protective shoe[(OR = 2.6;95% CI:2.2–7.3), P = 0.01], and children infected with tungiasis[(OR = 5.6;95% CI:0.3–7.63),P = 0.001] were found to be significantly associated with the prevalence of intestinal parasitic infection (Table 2). Whereas, mother’s education, gender, mother’s occupation and knowledge of mother how child gets parasitic infections didn’t show variation therefore they weren’t analyzed further.

Based on the above facts, children who were infected with tungiasis recurrently (three times per month) were found to be three times [(AOR = 3.1; 95% CI: 1.1–6.6), *P* < 0.001] more likely to be infected with intestinal parasites than other children who infrequently infected by tungiasis. Similarly, the children, who cared for defecting themselves were three times [AOR = 3.1; 95% CI: 1.1–5.5), *P* = 0.001] more likely to be infected with intestinal parasitic infection as compared to the children being cared for defecting by their mothers. Likewise, children who frequently eat raw/undercooked vegetable were more likely to be infected with intestinal parasitic infection as compared to the children who eat cooked vegetables and boiled milk [(AOR = 1.3; 95% CI:1.4–3.3), *P* < 0.001]. The history of geo-phage, children who were eat soil five times [(AOR = 4.7; 95% CI: 2.0–10.4), *P* = 0.001] more likely to be infected with intestinal parasitic infections as compared to those who were seldom to do so (Table 4).

**Table 4:** Multivariate analysis of factors associated with IPI among under-five children live in and around Haro Dumal Town, Bale Zone, Ethiopia, 2018

Risk factors	% positive for IPI	Crude OR 95%CI	*P*-value	AOR	*P*-value
Eat under-cooked/ raw vegetable					
Yes	125 (46.5)	1.5 [0.9–3.0]	0.001	1.3 [1.4–3.3]	<0.001
No	91 (31.2)	1.0	–	1.0	–
History of excessive crying					
Yes	170 (78.7)	2.4 [1.0–4.4]	0.01	2.1 [1.1–4.1]	0.014
No	46 (21.3)	1.0	–	1.0	–
Finger nail trimming					
Yes	141 (65.3)	1.9 [1.1–2.6]	0.01	1.6 [0.8–4.1]	0.02
No	75 (34.7)	1.0	–	1.0	–
Geophage					
Yes	115 (53.2)	5.2 [2.1–11.3]	0.01	4.7 [2.0–10.4]	<0.001
No	101 (46.8)	1.0	–	1.0	–
Latrine care					
Mother	58 (26.9)	0.9 [0.5–6.6]	0.61	1.0	0.86
Self	157 (72.7)	4.7 [1.6–12.5]	0.001	3.1 [1.1–5.5]	0.001
Others	1 (0.4)	–	–	–	–
Wear protective shoe					
No	132 (61.1)	2.6 [2.2–7.3]	0.01	2.3 [2.0–4.8]	0.01
Yes	84 (38.9)	1.0	–	1.0	
Child infected Tungiasis/ month					
1 time	3 (3.2)	1.0	–	1.0	–
2 times	43 (46.2)	1.9 [0.4–3.9]	0.01	1.6 [1.0–5.1]	<0.001
≥ 3times	47 (50.6)	5.6 [0.3–7.3]	0.001	3.1 [1.1–6.6]	

*IPI* intestinal parasitic infection, *AOR* Adjusted odd ratio, *OR* Odd Ratio, *CI* Confidence Interval, *P*-value of X^2^ test of the trends

## Discussion

Intestinal parasitic infections caused by helminths and protozoans remain a major public health problem among under-five children living in and around Haro Dumal Town dwellers, thereby the infection possibly contributes to socioeconomic catastrophe and impedes community health. This study revealed that the prevalence of intestinal parasitic infection was 38.5%and such high prevalence has been consistently reported by a number of studies conducted among under-five children live in vulnerable rural communities of Ethiopia. On the other hand, the present study showed very much higher prevalence of intestinal parasitic infections compared to the study conducted in Yadot primary school children of South Eastern Ethiopia 26.2% [12] and Dera district, South Gonder 20.9% [19]. Other studies conducted in many developing countries also agree with the current study that the prevalence of soil transmitted parasites is still high compared to WHO recommending and guideline [4, 11, 14, 16].

This finding showed that the prevalence of intestinal parasite was lower than the studies conducted among children in northwestern Ethiopia; 58.3% [1], Wondo Genet, Southern Ethiopia 85.1% [3]. The significantly low prevalence of parasitic infections observed in this study might be due to geographic difference, awareness to control intestinal parasitic infection and route of transmission. Moreover, health education provided by health extension workers is an important factor influencing individual’s attitudes on the prevention of intestinal infections and child care for their family [2, 5].

This study also revealed that under-five children have been susceptible to parasitic infection by helminthic and the availability of the sources of infection. Many studies showed that selective deworming has been playing a pivotal role in reducing prevalence of geo-helminthic parasites such as *T.trichuria, A.lumbricoides* and hookworm provided in this age group [9, 16, 17]. In contrast to this, the prevalence of geo-helminthes such *A.lumbricoides*, hookworm, *T tricuria*and *E. vermicularis* was 13.4, 14.4, 13.9 and 8.8% respectively. This shows that, there were no and/ or low coverage of the recent deworming programme in the study district or there were the possibility of having been re-infected after a period of deworming. Hence, there is the need for urgent intervention, training of health workers with a focus on prevention and control of parasitic infection, launching mass deworming and accessing health facilities [6, 12]. It is initiated us to evaluate the effectiveness and efficiency of the treatment provided for geo-helminths [8]. This agreed with the WHO strategy on eliminating soil-transmitted helminthes through protective chemotherapy in order to reduce child mortality from neglected parasitic infection to attain sustainable health developmental goals [6, 7, 13, 14].

The overall, prevalence of intestinal protozoan affection among under-five children was 25.5%. The prevalence of *E.histolytica/dispar* and *G. lambia was* 15.3 and 10.2% respectively. They had been continued to the principal intestinal human pathogenic protozoan among under-five children. This finding substantiates previous studies that *E. histolytica/dispar* and *G. lamblia* were predominant intestinal protozoan affect under-five children with gastroenteritis [8, 9]. This study argues that the prevalence of intestinal protozoan infections was ranging from 2.0–7.0% in developed countries and 20.0*–*30.0% in most developing countries [15]. Another study done in Nigeria and Tanzania confirmed that intestinal protozoa are a public health problem [16, 20]. Indeed, studies done in Ethiopia showed that almost all under-five children were infected with at least one of the two pathogenic intestinal protozoa [1–3, 12, 19]. Many researchers argued that in developing counties, where there is poor sanitation, untreated water and inadequate drainage systems and poor healthcare, those parasitic infections caused socioeconomic devastation and hampered healthcare in rural vulnerable communities [5, 6, 8]. ThereforeMoreover, they recommended that effective poverty reduction programmes preventive measure for ecto-parasites, promotion of mass deworming, health education and accessing health care facilities as the major controlling and reducing transmission of intestinal parasite carriage where the prevalence is high [9, 18]. Therefore, Those children who live in vulnerable rural communities need top urgent attention for preventing and control of intestinal parasites to attain sustainable health development goals and save millions of under-five children in developing countries.

## Conclusions

This study revealed that the overall prevalence of intestinal parasitic infection is high and needs a great attention to reduce and control transmission. Hence, health education, improving sanitation, provision of safe drinking water, control of ecto-parasites such as t*ungiasis* and mass deworming are essential to prevent and control IPIs in vulnerable rural communities.

## Data Availability

All data and materials of this work will be available from the corresponding author on request.

## Abbreviations

*A.lumbricoide*: *Acaris lumbricoide*
*E. histolytic*: *Entameba histolytica*
*G. lamblia*: *Gardia lamblia*
*H.nana*: *Hylonnopus nana*
IPI: Intestinal Parasitic Infection
*S.stercoralis*: *Strongloide stercoralis*
*T. saginata*: *Tenia saginata*
*T. trichiura*: *Tricurius tricuria*
